# Secondary prevention after acute and chronic coronary syndromes: are we still not there?

**DOI:** 10.1007/s12471-025-01936-5

**Published:** 2025-02-13

**Authors:** Michiel Voskuil

**Affiliations:** Department of Cardiology, University Medical Centre Utrecht, Utrecht, The Netherlands

Over the last decades, management options for acute and chronic coronary syndromes (ACS and CCS) have evolved enormously, including the availability of contemporary percutaneous coronary intervention (PCI) techniques. Consequently, in-hospital mortality after ACS has been reduced to approximately 5% [1]. Nevertheless, about 20% of ACS survivors experience an ischaemic cardiovascular event within 2 years after ACS and 5‑year mortality is still around 20% [2]. Besides the percutaneous treatment of coronary artery disease (CAD), secondary prevention in these patients has become one of the cornerstones of cardiovascular care. The treatment of the pathophysiological mechanisms behind cardiovascular disease, such as progression of lipid plaques, endothelial dysfunction, platelet aggregation and plaque rupture, remains crucial to further reduce morbidity, mortality and finally long-term healthcare costs.

Secondary prevention strategies target several risk factors, including hypertension, dyslipidaemia, diabetes, smoking, physical inactivity and poor diet, which all contribute to ongoing atherosclerotic progression and recurrence of ischaemic events. Given the multifactorial nature of CAD, the approach to secondary prevention must be equally comprehensive, involving pharmacological treatments, lifestyle modifications and, foremost, regular monitoring. However, as shown in the EUROASPIRE V registry, implementation of secondary prevention guidelines in Europe still needs considerable improvement. It demonstrated that 19% of patients with verified coronary artery events or interventions still smoked (of whom 55% was a persistent smoker), 38% were obese, and only 66% were physically active. In addition, 42% still had blood pressure ≥ 140/90 mm Hg (≥ 140/85 mm Hg if diabetic), 71% had low-density lipoprotein cholesterol ≥ 1.8 mmol/l (≥ 70 mg/dl), and 29% reported having diabetes. The reasons for not achieving secondary prevention goals are complex and multifactorial. They include incomplete or inconsistent follow-up, inadequate education about the importance of secondary prevention, psychological factors such as depression or anxiety and physical factors such as limited mobility or comorbidities.

In the current issue of the *Netherlands Heart Journal*, Woelders and co-workers describe the design and rationale of the South-East Netherlands Heart Registry (*Zuid-Oost Nederland Hart Registratie*, ZON-HR) [3]. They aim to improve secondary prevention in patients undergoing PCI, focusing mainly on antiplatelet treatment in a personalised fashion. For this purpose, they will use the Netherlands Heart Registry (NHR), a national database to which all interventional centres must submit data on patient and procedural characteristics and follow-up after PCI. However, the registration of parameters by the NHR is limited and mainly focuses on mortality and the need for revascularisation. A consortium of 4 PCI centres in the southeastern region of the Netherlands has now created an extended version, to be able to gather a more complete overview of the quality of secondary prevention and personalised medicine.

The investigators will include all patients from the NHR database who underwent PCI in their region, with a follow-up duration of 1 year. Only in patients regarded as high ischaemic risk based on presentation and patient characteristics, 2‑year follow-up data will be collected. Personalised secondary prevention post-PCI will be pursued using a risk stratification strategy for bleeding and thromboembolic risks to guide antiplatelet therapy. To further enhance personalised treatment, the investigators promote active screening for *de novo* or undertreated diabetes by measuring HbA_1C_ values. Furthermore, monitoring of cholesterol management during out-patient visits is stimulated. Outcomes including complications at 1 month, 1 year and 2 years after PCI are collected using digital questionnaires. Patient-reported outcomes are verified by electronic health record research, and laboratory results are gathered and subsequently registered in the database.

In the light of the low percentage of patients reaching treatment targets for secondary prevention, the initiative of the colleagues of the ZON-HR Consortium is praiseworthy. Still, some comments can be made. First, they use a protocol for antiplatelet treatment based on patient and procedural risk factors. To a large extent, they use the European guidelines as a reference for this algorithm. They also suggest the treatment of patients undergoing a complex PCI with dual antiplatelet therapy for 12 months. However, the European Society of Cardiology gives this only a class IIb recommendation, with a level of evidence B. [4] Evaluating the outcomes of this subset of patients will therefore be quite interesting.

Moreover, the investigators do not give a recommendation on the use of other pharmacological therapies proven to be effective in secondary prevention, such as angiotensin-converting enzyme inhibitors, angiotensin receptor blockers and sodium-glucose co-transporter 2 inhibitors. Furthermore, they do not offer any recommendations for post-PCI lifestyle modifications (e.g. smoking, diet, exercise).

## Conclusion

We are still not there yet. Cardiologists must commit to providing personalised care and intensifying patient guidance in a broad manner, to achieve our goals more effectively. Particularly important is strengthening the collaboration with patients and other healthcare providers (e.g. general practitioners and dieticians) to promote secondary prevention and support it as an integral part of recovery. Initiatives such as ZON-HR help in this effort, in conjunction with other Dutch initiatives, for example, the FORCE-ACS registry [5]. However, a more diverse and broadly supported programme is needed to significantly improve the percentage of patients reaching lifestyle and treatment targets, to improve the outcomes of this large and still growing patient population.
